# Evaluation and Acceptance of an Electric Toothbrush Designed for Dependent Patients

**DOI:** 10.7759/cureus.15372

**Published:** 2021-06-01

**Authors:** Virginia Prendergast, Kristina M Chapple

**Affiliations:** 1 Neurosurgery, Barrow Neurological Institute, Phoenix, USA; 2 Trauma/Acute and General Surgery, St. Joseph’s Hospital and Medical Center, Phoenix, USA

**Keywords:** caregiver, electric toothbrush, hospitalized, hygiene, icu, mouth, oral care, suction

## Abstract

Introduction: A key barrier to standardizing evidence-based oral health protocols for highly dependent patients is the lack of validated and accepted oral health products designed specifically for use by caregivers. This study compared preferences by users of a novel electric toothbrush and a manual toothbrush in a health care setting.

Methods: We prospectively enrolled health care providers as volunteers. Volunteer brushers completed simulated tooth brushing sessions of mock-intubated and non-intubated volunteer brushees with both toothbrushes. Volunteers rated different domains of toothbrush preference in an anonymous, optional survey.

Results: A total of 133 health care providers volunteered (123 brushers [providers brushing teeth] and 10 brushees [those having their teeth brushed]). The novel electric toothbrush received significantly higher positive ratings than the standard hospital-issue manual toothbrush in all domains that we surveyed: ease of use, thoroughness, safety, shape and size of the brush head, overall cleanliness, time requirements, and efficiency (p<0.001). Importantly, due to the integrated light and suction of this electric toothbrush, brushers completed more sessions without setting down the toothbrush with the electric toothbrush than with the manual toothbrush (75.4% vs 36.4%; p<0.001).

Conclusions: Integrating a lighted electric brush with suction into the caregiver’s armamentarium as an evidence-based tool is warranted and should be evaluated in terms of patient outcomes.

## Introduction

Oral care of hospitalized patients is an emerging area of nursing research and practice. Despite growing awareness, information is lacking on product acceptance and efficacy for hospitalized and dependent patients. Perhaps most importantly, evidence-based oral hygiene products embraced by caregivers need to be developed and deployed throughout hospitals, rehabilitation centers, and long-term care facilities to improve the fidelity to oral care protocols.

Several surveys of intensive care unit (ICU) nurses provide evidence of the growing interest in oral health care of hospitalized patients. In a survey of 218 ICU nurses across Israel, oral care was ranked as a high priority [1]. However, the authors concluded that evidence-based oral health care was not always provided, and further education was required. Authors of surveys in the United States and Sweden similarly concluded that the best practices are not always known or followed, and further research and training are needed [2-4].

The need for standardized, evidence-based products to provide oral care in hospitals will increase as the population ages. The United Nations estimates that the older population (over 60 years) will double between 2017 and 2050, reaching an estimated two billion people [5]. Periodontal disease, tooth caries, and tooth loss are more prevalent in older people, and older people are more likely to be admitted to a hospital or to be otherwise dependent upon caregivers to provide oral health care [6]. Several subgroups of patients are also at risk for preexisting periodontal disease, including those with stroke [7] or dementia [8] and patients from residential care [9].

Patients who are highly dependent upon health care providers for assistance with activities of daily living, including those with stroke or head trauma and patients who are in a coma or receiving mechanical ventilation, require additional consideration regarding oral hygiene. In addition, long-term weakness, paralysis, and dysphagia pose challenges to maintaining good oral health [7,10-15]. Intubated patients also present a unique challenge because the endotracheal tube is a physical and visual barrier to providing oral health care.

Over the last 20 years, the association between poor oral health and pathogenic oral bacteria in hospitalized patients and nursing home residents has been clearly established [16,17]. Additionally, a growing body of evidence suggests a strong correlation between aspiration pneumonia and pathogenic oral bacteria, which can be inhaled (aspirated) into the lungs in small droplets of oropharyngeal secretions [18]. Hospital-acquired pneumonia, including ventilator-associated pneumonia, is a major cause of morbidity and mortality [18,19]. In 2003, a comprehensive review of 24 cohort studies reported a significant association between pneumonia and dental plaque or dependency in oral hygiene [20], and studies have shown that enhanced oral health protocols reduced lower respiratory tract infection [21], hospital-acquired pneumonia [22], and ventilator-associated pneumonia [23,24]. However, recent reviews reported that insufficient evidence exists to conclude that enhanced oral health protocols can reduce respiratory infections and pneumonia [18,25], possibly due to the lack of standard, evidence-based protocols and products.

We developed the evidence-based Barrow Oral Care Protocol to improve and standardize the oral health care provided at our institution. The protocol and supporting evidence have been described in detail [23]. An electric toothbrush specifically designed for dependent patients was conceived by comparing and contrasting the efficacy of evidence-based products and user feedback. During follow-up discussions, nurses provided feedback on their beliefs about the most efficient and effective ways to provide oral care. Themes such as needing to set a toothbrush down multiple times when providing care, difficulty viewing all areas of the mouth in intubated patients, and needing suction to ensure water did not drip into the patient’s throat and lungs emerged. This feedback led to the idea that an electric toothbrush designed specifically for health care providers to use with dependent patients would help overcome the reported barriers and improve fidelity to oral care protocols by caregivers while decreasing the environmental impact of single-use, disposable plastic products.

A novel battery-powered toothbrush was developed and refined through multiple rounds of prototyping. The brush features a built-in light and a connection to suction, enabling teeth cleaning with one hand and removing the need to set the toothbrush down when using suction. The toothbrush was designed with a rotating, oscillating brush head, shown to be superior to a manual toothbrush in removing plaque and reducing gingivitis [26]. Additionally, the brush head is removable, which allows the base to be cleaned and reused throughout the patient’s hospitalization, ensuring infection control and producing less biohazard waste than current standard-of-care single-use products.

As preference and acceptance drive the rate at which a new idea will spread [27], we designed a study to gather feedback from end-users (i.e., oral health care providers) after using the novel electric toothbrush in a simulated real-world setting. The primary goal of the study was to measure user acceptance and preference of the novel battery-powered toothbrush compared to a manual toothbrush for use in a health care setting for both intubated and non-intubated patients. We also collected demographic information regarding health care roles and experience levels and end-user feedback regarding safety, effectiveness, and discomfort.

## Materials and methods

Design and setting

This study was prospectively conducted at three urban hospitals in the southwestern United States with approval from the St. Joseph’s Hospital and Medical Center Institutional Review Board (Phoenix, AZ). The study was conducted in the Simulation Center of one of the participating hospitals during weekday and weekend shifts. The Simulation Center is located within the hospital and is used as the main educational center for health care professionals. Participants were recruited as either brushers (to brush teeth) or brushees (to have their teeth brushed) using both a manual and the novel electric toothbrush. Brushers and brushees participated as volunteers in scheduled simulations during nonwork hours.

Brushers began by reading typed instructions that detailed specified tasks: brush teeth, use suction, and use light. All brushers wore masks with eye shields and gloves. Brushers who routinely work in ICUs completed their session with endotracheal tubes taped to the patients’ mouths to mimic intubated patients. The novel electric toothbrushes were prototype versions with a nondetachable head and used once per volunteer brushee. Manual toothbrushes were standard hospital-issue toothbrushes used for inpatients. The brushers manually added gel or toothpaste to the toothbrush.

Brushees were asked to lie on a hospital bed for the brushing simulations. Following the initial brushing simulation, the brushee was offered light snacks and water for comfort and to add oral debris before the next brushing simulation. This step enhanced the opportunity to assess the cleaning efficacy of the toothbrush by simulating the brushee having eaten a meal. Regardless of which brush was used, the brushing time was restricted to a minimum of one minute and a maximum of two minutes. Brushee participation time did not exceed one to two hours per simulation session to minimize the effects of repeated tooth and gum brushing.

Measures

Optional surveys were designed for the brusher and brushee to complete after each brushing session. The brusher survey consisted of demographic items, work experience items, and ratings of the different aspects of the brush and the experience of using the brush. Survey items in addition to the demographic questions are shown in Table 1.

**Table 1:** Brusher survey items *Items were rated on a continuous Likert scale from one to nine, with one being the most negative to nine being the most positive, unless otherwise specified. †Likert, 1-5. Abbreviation: NA, not applicable.

Rating	Likert Scale (1-9)*
Item	
I was able to clean the mouth easily.	Disagree to agree
I was able to clean the mouth thoroughly.	Disagree to agree
I was able to clean the mouth safely.	Disagree to agree
Usefulness of light	Not valuable to valuable
Effectiveness of suction	Not effective to effective
Shape and size of brush head	Awkward to convenient
Overall does a good job	Disagree to agree
Is too time-consuming to use	Disagree to agree
Is efficient	Disagree to agree
Is comfortable to hold	Disagree to agree
How important is oral health care in the hospitalized setting?	Not important to important^†^
Multiple-choice	
How many times did you set the brush down?	0, 1, 2, 3, or more
Assuming equal cost, which brush would you prefer for your patients?	Electric or manual
Open-ended feedback	NA
Provide any feedback regarding the use of this electric toothbrush used with patients	NA
List three words to describe your experience with the electric brush	NA

Participants were asked to rate items on a nine-point continuous Likert scale, with one reflecting disagreement or negative ratings and nine reflecting agreement or positive ratings. The brushee survey consisted of demographic items and nine-point Likert-type items to allow for ratings of comfort level and usefulness of suction. Participants were asked to provide open-ended feedback at the end of both the brusher and brushee surveys.

Data management and statistical analysis

At the planning stage, we aimed to recruit 100 volunteer health care providers to achieve 91% power to detect a mean of paired differences of 1.0 and an estimated standard deviation of 2.0, using a two-sided t-test with a significance level of 𝛼=0.05. Data were summarized using frequencies with percentages, means with standard deviations, and medians (25th and 75th) percentiles. Mann-Whitney U tests were used to compare medians between groups. McNemar’s test was used to compare the proportion of dichotomous outcomes for paired samples. Data were analyzed using the SPSS Statistics for Windows, version 25.0 software (IBM Corp., Armonk, NY, USA).

## Results

The study sample consisted of 133 volunteer health care providers (123 brushers and 10 brushees) who worked in large urban medical centers or long-term acute care centers where vulnerable patients were routinely unable to perform their oral hygiene. The majority of brushers reported working in an urban setting (n=95, 77.2%) and in the ICU (n=78, 63.4%), and many had cared for patients for more than 10 years (n=47, 38.2%) (Table 2).

**Table 2:** Brusher demographics

Characteristic	Frequency (N=123), N (%)
Facility	
Urban hospital 1	79 (64.2)
Urban hospital 2	16 (13.0)
Long-term acute care facility	26 (21.1)
Unknown	2 (1.6)
Primary worksite	
Intensive care unit	78 (63.4)
Hospital floor	16 (13.0)
Rehabilitation unit or skilled nursing facility	4 (3.3)
Long-term acute care facility	24 (19.5)
Other	1 (0.8)
Years cared for patients	
Less than two years	18 (14.6)
Two to five years	31 (25.2)
Six to nine years	27 (22.0)
Greater than 10 years	47 (38.2)
Home use	
Electric brush	83 (67.5)
Manual brush	40 (32.5)

Brushers who did not work in an ICU reported working in a long-term acute care facility (19.5%), on the hospital floor (13.0%), in a rehabilitation or skilled nursing facility (3.3%), or other (0.8%). Most brushers, regardless of their experience level providing oral health care for vulnerable patients, reported using an electric toothbrush at home (n=83, 67.5%).

A comparison of the survey results between the electric and manual toothbrushes for ease of use, thoroughness, safety, shape and size of the brush head, overall cleanliness, time requirements, and efficiency demonstrated a statistically significant advantage for the electric brush (p<0.001) for each of the seven assessed parameters (Table 3).

**Table 3:** Likert score* results reflecting brusher responses related to safety, effectiveness, comfort, and ease of use of the toothbrushes *Items were rated on a continuous Likert scale from one to nine, with one being the most negative to nine being the most positive. Abbreviation: IQR, interquartile range.

Parameter	Electric, median (IQR)	Manual, median (IQR)	P-value
Ease of use	9 (8-9)	7 (5-9)	<0.001
Thorough	9 (8-9)	7 (4-9)	<0.001
Safety	9 (8-9)	7 (4-9)	<0.001
Shape and size	9 (8-9)	7 (3-9)	<0.001
Overall cleanliness	9 (8-9)	7 (4-8)	<0.001
Time consuming	1-2 (1)	5 (1-8)	<0.001
Efficient	9 (8-9)	6 (3-8)	<0.001

Opinions of, preference for, and acceptance of the electric toothbrush versus a manual brush are summarized in Table 4.

**Table 4:** Results of brusher surveys on opinions, preferences, and acceptance levels of a novel battery-powered toothbrush versus a manual brush

Item	Frequency, N (%)
How important do you feel oral care is for hospitalized patients	N=123
Very important	99 (80.5)
Important	20 (16.3)
Moderately important	4 (3.3)
Minimally important	0 (0)
Not important	0 (0)
Which brush do you think is better for the environment	N=121
Electric	115 (95.0)
Manual	6 (5.0)
Assuming equal cost, which brush would you prefer for patients	N=122
Electric	117 (95.9)
Manual	3 (2.5)
Unsure	2 (1.6)

A significant proportion of brushers (99/123, 80.5%) reported they consider oral care to be very important for hospitalized patients. Nearly all brushers believed electric toothbrushes are more environmentally friendly (115/121, 95.0%). All but five of 122 brushers (4.1%) preferred the electric toothbrush if cost were not a consideration.

**Figure 1:**
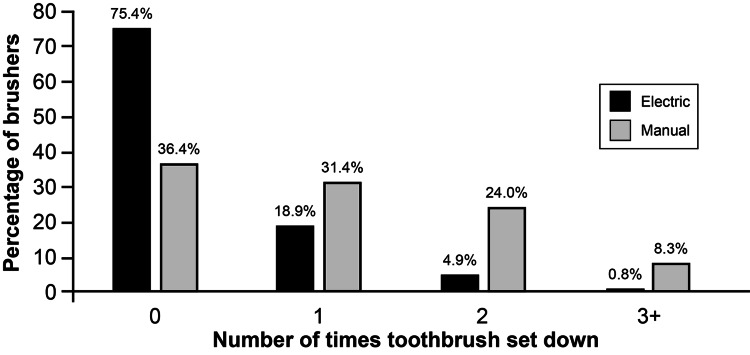
Percentage of times a brusher reported setting the toothbrush down during a brushing session. Used with permission from Barrow Neurological Institute, Phoenix, Arizona.

Most brushers reported favorable opinions related to the operational capacity of the electric toothbrush. On the nine-point Likert scale, 98.3% (116/118) rated the light as valuable (ratings of six to nine), with 78.0% (n=92) assigning the light a maximum rating of nine. Suction was rated as effective (ratings six to nine) by 95.9% (115/120) of brushers. Brushers were significantly more likely to complete the brushing session without setting down the electric brush 75.4% (92/122) compared to the manual brush 36.4% (44/121), p<0.001 (Figure 1).

Seventy-nine open-ended comments were written regarding the brushers’ experiences with the brushing session. There were 17 comments on suction, of which 16 were related to the suction strength being used at the simulation station. Fourteen brushers explicitly commented that the light was a positive feature on the brush, and 13 comments indicated approval of the brush design for its ease of use. Nine comments suggested it is beneficial to hold the light, suction, and brush as one and within one hand, five brushers commented this brush could provide an important addition to patient care, and one comment noted the brush felt bulky. Other comment themes included general feedback (n=20) such as “I want to see this brush again soon,” “awesome toothbrush,” and “excellent innovation.”

A total of 10 staff members had their teeth brushed during 145 sessions. The electric toothbrush was used in 81 (55.9%) sessions, and 93 (64.1%) sessions were completed with mock intubation. Two brushees were men, and eight were women. Seven reported using an electric brush at home. On a Likert scale of one to nine (one, no agreement; nine, complete agreement), nine brushees reported significantly increased comfort during their session when the electric brush was used (ratings, eight or nine) than the four brushees with a manual session (ratings, three to seven); p<0.001). There were 81 ratings by brushees on the effectiveness of the suction. All but one rating was at the midpoint of the scale at five or higher, and 58/81 (72%) ratings were eight or nine, reflecting the most positive rating of suction effectiveness.

Brushees provided a total of 61 comments on the sessions. The majority of comments (n=34) were essentially a critique of the brusher with statements such as “nurse was rough” or “very gentle.” Seventeen comments were related to suction, with 15 related to the appropriateness of the suction and two suggesting suction at the simulation center should be stronger. Seven general comments were given, and all were positive, suggesting the electric toothbrush was superior to the manual brush. One brushee commented that the brush felt big for her mouth.

## Discussion

Nurses are uniquely positioned to drive change and innovation in health care, especially for specific patient populations, health care procedures, and products they interact with daily. In 2019, Albert [28] described the need for nurse innovators to incorporate health care innovations into nursing practice, including those for oral health care management. Albert details the critical nature of nurse leaders serving as innovators to propel the term “innovation” to the forefront of the practice and to make it a part of the foundational work performed in nursing programs.

The nursing innovation mindset at our institution provided the foundation where the director of advanced practice nursing (author V.P.), with the help of a registered dental hygienist, developed an evidence-based oral health care protocol to bring principles of oral hygiene proven to be effective in the general population into the health care setting [23]. During the development of the protocol, themes began to emerge from oral care providers (i.e., nurses). They reported that it was difficult to know how much pressure to use and difficult to maneuver around the patient’s mouth with a pediatric toothbrush, which is still the standard issue toothbrush in most hospitals. In addition, research demonstrates that an electric toothbrush with an oscillating rotary head is superior to manual toothbrushes for removing dental plaque and improving gingival health [26]. Thus, an electric toothbrush was added to the protocol, which solved the dexterity and pressure challenges. The protocol with an electric toothbrush improved oral health in ICU patients, reduced ventilator-associated pneumonia, and decreased costs at our institution [11,23]. However, nurses tasked with performing the oral health care cited lack of visibility due to little or no light and the need to set the toothbrush down to suction the patient’s mouth as challenges.

This front-line feedback from hands-on users drove the design of an electric toothbrush that contained an LED light and a suction port. With the help of an engineer and a registered dental hygienist, a toothbrush was manufactured and went through eight rounds of prototyping with prospective brushers (i.e., nurse providers of oral health care).

For this study, we recruited health care professionals who routinely provide oral care to patients. We designed a simulated health care setting, where volunteers participated as mock end-users of the novel electric toothbrush (i.e., brushers) or mock patients (i.e., brushees). We designed an optional anonymous survey for both the brusher and the brushee (Table 1) to measure the acceptance and preferences of the volunteer brushers on a nine-point continuous Likert scale and to collect demographic and open-ended feedback.

In agreement with previous studies [1-3], a large majority (96.8%) of the brusher volunteers in our study agreed that oral health care is important or very important for hospitalized patients. The brusher volunteers reported a significantly higher preference for the novel electric toothbrush than the manual toothbrush in every domain surveyed, including ease of use, thoroughness of cleaning, and efficiency (Table 3).

The novel features of the electric toothbrush include light and suction. Brushing a patient’s teeth is one of the few tasks that a nurse is expected to perform with insufficient light. A light that allows the health care provider to visualize inside the mouth cavity enables targeted brushing and the detection of problems. Indeed, a large majority (98%) of the brushers rated the light as valuable (ratings of six to nine). Suction is a standard in most hospital rooms; however, a provider must use a secondary suction device or set down the standard toothbrush to use the suction. The novel electric toothbrush addresses this problem by integrating the suction and the toothbrush into a single device. Over 95% of brushers rated the suction as useful (ratings of six to nine). Furthermore, the number of brushing sessions that the brusher did not have to set the brush down at all was more than double for the electric toothbrush compared to the manual toothbrush (Figure 1).

In the final overall survey question, over 95% of volunteer brushers reported that they would prefer the electric toothbrush over the manual toothbrush, assuming comparable cost. As preference and acceptance drive the rate at which a new idea spreads, a theory detailed in Everett Rogers’ book Diffusion of Innovations [27], we assessed the use of the novel electric toothbrush with early adopters. The study results provide evidence that the novel electric toothbrush could achieve wide adoption throughout the health care community. Overall, the novel electric toothbrush received an overwhelmingly positive response. Participants indicated their eagerness to use it in clinical practice.

The strengths of this study include its relatively large sample size and the setting, which simulated patient scenarios with the brushee lying in a hospital bed and the brusher using the prototype electric toothbrush. The weaknesses include volunteer brushees instead of actual patients, simulated placement of endotracheal tubes, and self-report data. Data were obtained from fewer brushers (n=123) than the number of brushing episodes (n=145). Additionally, because the survey was optional, there was no way to know the opinions of the brushers who decided not to complete the study.

## Conclusions

As front-line clinicians, we observed a pressing need for a standardized, evidence-based oral health care protocol to improve oral health and patient outcomes in our ICU. This scrutiny led to the development of a novel electric toothbrush designed to overcome specific barriers - a lack of visibility inside the patient’s oral cavity and the need to put the toothbrush down to apply suction. The brushers placed a high value on the integrated light and suction of the electric toothbrush.

We designed this health care simulation study and optional anonymous surveys to gather end-user feedback and assess the level of user acceptance of this unique and innovative electric toothbrush. In all domains surveyed, this novel electric toothbrush received significantly higher positive ratings than the standard hospital-issue manual toothbrush. Nearly all volunteer brushers preferred the innovative electric toothbrush over the manual toothbrush, assuming comparable cost. The integration of an electric brush into the caregiver’s armamentarium as an evidence-based tool is warranted. Future research should evaluate the impact of the electric brush on patient outcomes, including hospital-acquired infections.
